# A trade‐off in vital rates for a large carnivore inhabiting an anthropogenic landscape and a protected island ecosystem

**DOI:** 10.1002/eap.70289

**Published:** 2026-08-02

**Authors:** Monica R. Cooper, Andrew Edwards, Kassandra Arts, Ronald Nordin, Jonathan N. Pauli

**Affiliations:** ^1^ Department of Forest and Wildlife Ecology, University of Wisconsin, Madison Madison Wisconsin USA; ^2^ Red Cliff Band of Lake Superior Chippewa Treaty Natural Resources Department Bayfield Wisconsin USA; ^3^ Apostle Islands National Lakeshore Bayfield Wisconsin USA; ^4^ Present address: Native American Fish and Wildlife Society Northglenn Colorado USA

**Keywords:** Anthropocene, black bear, human food, life history, masting, *Quercus*, stable isotopes, *Ursus americanus*

## Abstract

Anthropogenic landscapes generally provide abundant human foods but increase mortality risk, which can alter vital rates for some species. Some species exhibit altered survival in anthropogenic landscapes, whereas others exhibit changes in reproduction. While changes in vital rates have been previously documented, the role of human food subsidies in vital rate trade‐offs is often overlooked, especially for large carnivores. Here, we explored how human food subsidies affect American black bear (*Ursus americanus*, *Makwa* [Ojibwe]) apparent survival, recruitment, and population growth rate. We compared bears in a protected island ecosystem featuring minimal risk of human‐caused mortality and little human food to a neighboring mainland population featuring relatively high human‐induced mortality from hunting but abundant human food subsidies. We estimated proportional diets of 105 bears using stable isotope analysis (δ^13^C and δ^15^N), assessed how landcover types in bear use areas influence diet, and explored how these variables affected bear vital rates. Island bears consumed almost entirely natural foods, principally hard mast, with virtually no human foods. Mainland bears consumed a diversity of items with nearly 1/3rd of their diet coming from human foods. Bear survival on protected islands increased with the amount of hard mast consumed, while survival on the mainland was not correlated with either diet or landcover. These populations also exhibited notable differences in vital rates: bears on the islands were characterized by high survival (>0.8 annual survival) but low recruitment (<0.2 annual recruitment), whereas bears on the mainland exhibited 37% lower survival (0.44 annual survival) but 35% (0.54 annual recruitment) higher recruitment. Our work suggests that anthropogenic landscapes, and notably food subsidies they provide, can lead to a trade‐off in vital rates by changing the risk‐reward landscape. Conversely, refugia that maintain natural trophic pathways can maintain intact food webs and ecosystem processes.

## INTRODUCTION

Food availability is a principal driver of animal population dynamics (Sinclair & Krebs, 2002). In general, animals face a trade‐off in allocating food resources between reproductive success and survival (Stearns, 1976). Reproduction is a plastic trait that often changes according to food availability (Williams et al., 2017). For example, gulls (*Larus* spp.) exhibit adaptive bet hedging breeding strategies, where food availability drives reproductive variables ranging from individual egg volume to overall clutch size (Oro et al., 2023). The effects of food availability on survival are typically less pronounced, especially for species with slow life histories (Chevallier et al., 2020). In arctic foxes, an increase in food can increase reproductive output while the lack of food reduces reproductive output even though survival remains unaffected (Chevallier et al., 2020). Yet these responses are not uniform across populations as other studies have found that arctic fox survival is related to food availability (Hiruki & Stirling, 1989). Furthermore, other species of mammalian carnivores exhibit strong changes in survival in response to food shortages (O'Donoghue et al., 1997; Peterson et al., 1998). Thus, the availability and variability of food resources can shape the vital rates of individuals and populations differently, even within the same species.

Humans have dramatically altered terrestrial ecosystems resulting in novel anthropogenic landscapes (Ellis & Ramankutty, 2008; Williams et al., 2016). Anthropization confers both risk and reward to species with some winning and some losing (Fisher & Burton, 2018; Suraci et al., 2021). Anthropogenic landscapes generally provide abundant sources of human food, and animals that exhibit dietary flexibility can benefit from increased resource availability (Manlick & Pauli, 2020; Newsome & van Eeden, 2017). The concentrated and predictable nature of human food can facilitate more efficient foraging compared to natural food resources, which often fluctuate (Yang et al., 2008). Consequently, human food subsidies can increase individual reproduction (Lee et al., 1986), reduce the age of primiparity (Wightman et al., 2022), increase survival (Prange et al., 2003), reproductive success (Picardi et al., 2024), and population densities (Fedriani et al., 2001). Anthropogenic landscapes featuring food subsidies can also present risks to animals and in extreme cases, act as an ecological trap where maladaptive behaviors can lead to population declines or extinctions (Moss et al., 2016; Schlaepfer et al., 2002). In other cases, animals confront increased mortality via compensatory recruitment (i.e., density dependent changes, often in response to harvest; Servanty et al., 2011; Villellas et al., 2015). Overall, species can exhibit nuanced demographic responses to humans and our subsidies. Understanding the potential trade‐offs that occur in response to the risks and rewards of human influence is essential for promoting species persistence in the Anthropocene.

American black bears (*Ursus americanus*, *Makwa* [Ojibwe]) are opportunistic foragers that consume a wide variety of resources (Merkle et al., 2013). Hard mast, soft mast, and animal matter are the principal diet items that bears rely on to satisfy high caloric needs in the American Midwest (Rogers, 1976). Summer and autumn food production, oak (*Quercus* spp.) mast in particular, can affect bear demography and vital rates (Costello et al., 2003; Reynolds‐Hogland et al., 2007; Rogers, 1987). Food shortages reduce juvenile survival and cub recruitment (Noyce & Garshelis, 1997), and in extreme cases lead to reproductive failure (Costello et al., 2003; Dobey et al., 2005; Jonkel & Cowan, 1971). Bears inhabiting anthropogenic landscapes expand their diets to include human food subsidies, especially during years of mast failure (Kirby et al., 2019; Lewis et al., 2014; Noyce & Garshelis, 1997). Human food can comprise up to 40% of bear diet (Hopkins et al., 2014; Kirby et al., 2017) and strongly influences bear vital rates (Ditmer et al., 2016). While human foods may not affect the number of cubs per litter (Morin et al., 2024), they can influence the age of primiparity and the proportion of females that reproduce annually (Bridges et al., 2011; Gould et al., 2021; Wightman et al., 2022). Yet, there are risks associated with consuming human foods. For example, bears in the Rocky Mountains, USA, consuming human refuse experienced higher adult mortality, which was predicted to lead to population decline (Lewis et al., 2014). Baiting for hunting typifies the risk‐reward trade‐off because these readily available calories simultaneously confer an implicitly high risk in the form of hunter harvest (Malcolm & Van Deelen, 2010). Overall, while human food subsidies appear to influence bear vital rates generally, it is not clear whether vital rate plasticity from consuming human foods could be a mechanism leading to a trade‐off in vital rates.

The Apostle Islands, *Wenaboozhoo ominisan*, is an archipelago of 22 islands in Lake Superior that lies off the coast of the Bayfield Peninsula of northern Wisconsin, where the Red Cliff Reservation, *Gaa‐miskwaabikaang*, constitutes approximately 60 km^2^. Bear densities on the mainland of Wisconsin (0.43 bears/km^2^; Kirby et al., 2017) and on the Apostle Islands (up to 0.88 bears/km^2^; Belant et al., 2005) are high, but it is unclear what role both natural and human food consumption play in these populations. Across Wisconsin, except for on the Apostle Islands (USDOI National Park Service Apostle Islands National Lakeshore, 2014), the use of intentional bear bait for hunting is common (Putman & Staines, 2004). There are over 15 million L of bear bait distributed annually in Wisconsin (Rees et al., 2014), and a bear home range can include up to 63 bait piles per year (Kirby et al., 2017). Foods in bait are restricted to non‐animal products but are often calorically rich, and feature items like donuts, candies, and cereals. In addition to bait piles, the mainland features human populations with associated agriculture and refuse that can be sources of human food. On the islands, hunting is legal but rare (Appendix S1: Table S1; zero harvest during this study), and so they feature little risk of human‐caused mortality and little human food. The mainland, on the other hand, features higher risk of human‐caused mortality (Appendix S1: Table S1; 0.08 bears hunted per km^2^ per year in GMU03 during this study) and abundant human food. The close proximity (separated by ~2 km) of the protected islands to the mainland provides a unique opportunity to explore how human food subsidies affect bear vital rates.

To test how the coupling of food subsidies and risk affect the vital rates of bear populations, we investigated survival and recruitment of bears as a function of diet and landscape. We estimated assimilated diets of bears and bear foods using stable isotope analysis (δ^13^C and δ^15^N) and quantified how landscape cover could influence bear diet to determine the origin of specific diet items. We then modeled how these variables affected bear apparent survival and recruitment. We hypothesized that bears inhabiting anthropogenic landscapes would consume more human foods and exhibit altered vital rates. Specifically, we predicted that mainland bears would rely heavily on human foods and that this reliance would be a function of landscape anthropization. We predicted that mainland bear apparent survival would decrease with human food consumption, and that mainland bears would exhibit low apparent survival, but high apparent recruitment ultimately resulting in a stable population (λ ≈ 1). In contrast, we predicted that bears inhabiting the islands would principally consume mast, which would increase as a function of oak landcover. We predicted that island bear apparent survival would increase with hard mast consumption, and that island bears would exhibit moderate survival, but that low apparent recruitment would ultimately lead to a stable or shrinking population (λ ≤ 1).

## METHODS

### Study area

The Apostle Islands are an archipelago located in southwestern Lake Superior in Wisconsin, USA. Twenty‐one of the 22 islands are within the Apostle Islands National Lakeshore which was established in 1970. Island sizes range from 0.08 to 40 km^2^; island distances from the mainland range from 1.48 to 23.83 km; and island elevations range from 8 to 147 m above sea level. The islands have a history of timber extraction starting in the late 1800s and concluding in 1955 (Judziewicz & Koch, 1993). Though forest composition changed, over 96% of the Apostle Islands remain forest habitat (Kraft et al., 2007, Figure 1). Maple‐yellow birch and northern hardwood forests dominate the islands (65%) followed by white‐cedar boreal conifer mesic forest (13%) and north‐central hemlock‐hardwood forest (<1%; Hop et al., 2010). Several islands have understory dominated by Canada yew (*Taxus canadensis*) due to a lack of deer irruption. Canada yew is rare on the mainland (Johnson et al., 2015). All terrestrial carnivores found on mainland Wisconsin are also present on the islands except for American badgers (*Taxidea taxus*) and striped skunks (*Mephitis mephitis*; Allen et al., 2018). On the islands, black bears (*Ursus americanus*) have the highest occupancy of any mammal (Allen et al., 2018).

**FIGURE 1:**
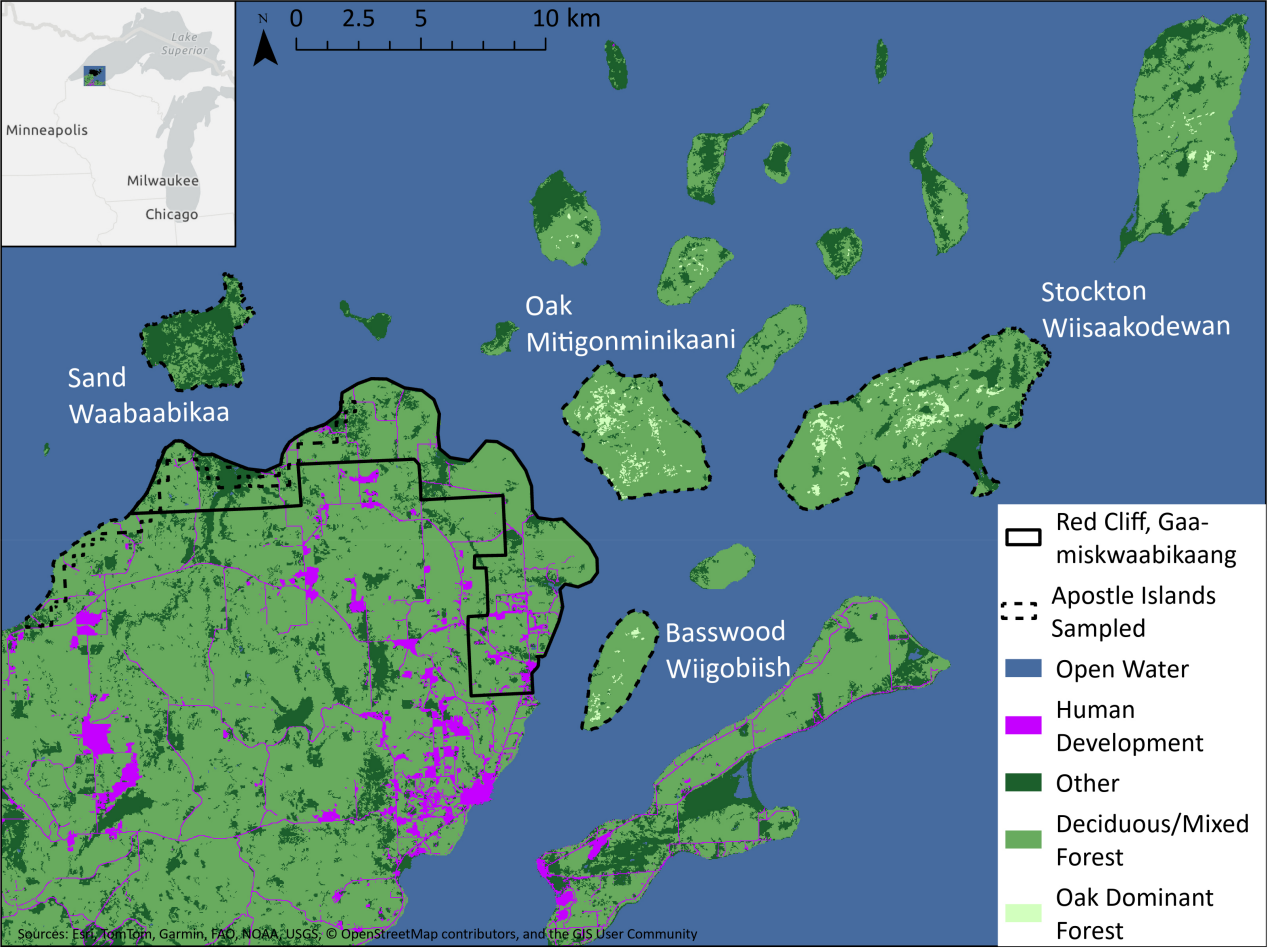
Map of landcover types used to analyze proportional landcover in use areas of black bears (*Ursus americanus*) in the mainland and Apostle Islands, Wisconsin, USA. The black lines outline the extent of sampling for this study within the Apostle Islands National Lakeshore and Red Cliff Reservation, *Gaa‐miskwaabikaang*.

### Stable isotopes

We collected bear hair noninvasively (Woods et al., 1999) across four islands where reproducing populations have been documented: Stockton (Wiisaakodewan), Sand (Waabaabikaa), Basswood (Wiigobiish), and Oak (Mitigominikaani) Islands. We also sampled along the adjacent mainland of Wisconsin within the Apostle Islands National Lakeshore and Red Cliff Reservation (Figure 1). We placed a single hair corral at the center of a 1.6‐km^2^ grid that we overlaid across the archipelago. This resulted in a total of 57 hair corrals (17 corrals on Stockton, 8 on Oak, 5 on Sand, 4 on Basswood, and 23 on the mainland). We collected genetic samples every 9–12 days for six sessions from May to August 2020–2022. We used 17 microsatellite markers to genotype and identify 136 individuals, 77 on the mainland and 62 on the four islands. Three individuals were detected on both the mainland and islands (for details see Cooper et al., 2024). We processed at least one stable isotope hair sample for each individual where we had remaining material. This resulted in stable isotope samples for 56 individuals on the mainland and 49 individuals on the islands. We collected common bear diet items including soft mast (*n* = 35; *Cornus* spp., *Ribes* spp., *Rubus* spp., *Vaccinium* spp., *Amelanchier arborea*, *Fragaria* spp., *Prunus* spp., *Aralia nudicaulis*, *Sambucus canadensis*), hard mast (*n* = 33; *Quercus* spp.) and ants and ant larvae (*n* = 13; *Formicidae*), white‐tailed deer muscle tissue (*n* = 19; *Odocoileus virginianus*), and beaver muscle tissue (*n* = 7; *Castor canadensis*) from the Apostle Islands and the adjacent mainland in summers of 2021, 2022, and 2023. The deer and beaver samples were collected from roadkill. We rinsed hair and soaked muscle samples with 2:1 chloroform:methanol solution. All samples were homogenized and then dried at 55°C for at least 72 h (Pauli et al., 2009). Analysis of stable isotopes was conducted using a PDZ Europa ANCA‐GSL elemental analyzer interfaced to a PDZ Europa 20–20 isotope ratio mass spectrometer (Sercon Ltd., Cheshire, UK). Results are expressed in parts per mil (‰) relative to the international standards of Peedee Belemnite (δ^13^C) and atmospheric nitrogen (δ^15^N) calibrated with internal laboratory standards. We included known diet items from previously published literature including bear bait (Kirby et al., 2017), corn (Ditmer et al., 2016), and human food (Newsome, Garbe, et al., 2015). We used digestible elemental concentrations of an average bear diet determined by Hopkins et al. (2012) and previously reported source specific trophic discrimination factors (TDFs; Appendix S1: Table S2). TDFs account for the effects of assimilation and excretion on the isotope value of the consumer, which can vary depending on type of tissue or animal species studied (Ben‐David & Flaherty, 2012). Due to the large variation of TDFs in the literature, we also ran mixing models with a single TDF (SD) value for a mixed diet omnivore in the mainland model (TDF_C_ = 2.0 [0.2]; TDF_N_ = 3.3 [0.2]; Stephens et al., 2023) and a C_3_ diet omnivore in the island model (TDF_C_ = 4.3 [0.4]; TDF_N_ = 3.2 [0.3]; Stephens et al., 2023; see Appendix S1). We calculated digestible elemental concentrations for samples collected in this study including animal matter, soft mast, and hard mast (Phillips & Koch, 2002).

To estimate bear proportional diet, we ran separate models for the islands and the mainland. We excluded three bears that made movements between the mainland and islands from mixing models. Since bear baiting is prohibited in the Apostle Islands National Lakeshore and bears have no access to corn fields on the islands, we included source groups for human food, hard mast, soft mast, and animal matter in models for island bears. For mainland bears, we included additional sources of corn and bear bait. We determined distinct isotopic food groups for analysis by K‐nearest neighbor (*p* < 0.05) and ecological relevancy (Rosing et al., 1998). We used a concentration‐dependent Bayesian multisource mixing model to estimate the relative proportions of prey in bear diet using *MixSiar* v3.1 (Stock et al., 2018; Stock & Semmens, 2016a). We used uninformative priors and ran three chains of 3,000,000 iterations, removing the first 1,500,000 iterations as burn‐in and thinning posterior samples to every 500th sample (Stock et al., 2018). We ran one model incorporating individual as a fixed effect to obtain mean diet estimates for individuals, including both residual and process error since we had multiple samples for many individuals (Stock & Semmens, 2016b). We ran a second null model, including both residual and process error for both the islands and the mainland to obtain overall population median and 95% credible interval diet estimates. We determined model convergence by Gelman–Rubin statistics (Brooks & Gelman, 1998; Gelman & Rubin, 1992). We aggregated the source groups bear bait, human food, and corn into a “human food” source group a posteriori for mainland models, using generalist priors (Stock et al., 2018). We directly compared this aggregated human food source group to the human food group from island models (Appendix S1: Figure S1).

### Landcover

We used the Vegetation Inventory and Map for the Apostle Islands to create a raster of oak (*Quercus* spp.) dominated forest with 10 m × 10 m resolution by aggregating oak specific layers (Northern Pin Oak Forest, Northern Red Oak—Sugar Maple Forest, and Jack Pine—Northern Pin Oak Forest; Hop et al., 2010). We used the Wiscland2 landcover dataset (30 m × 30 m resolution) to create a raster of oak (*Quercus* spp.) forest (Wisconsin Department of Natural Resources, 2016). To assess the relationship between anthropogenic landcover and diet on the mainland, we obtained landscape composition in 2021 from the National Land Cover Dataset (Dewitz & U.S. Geological Survey, 2021; Taylor, 2023). We created an anthropogenic land cover category by aggregating cover classes: developed open space, developed low intensity, developed medium intensity, developed high intensity, pasture/hay, and cultivated crops. We determined a use area for each bear by creating a buffer around detected locations with the radius of male and female bear home range on the islands (*M* = 2.46 km, *F* = 1.57 km) and the mainland (*M* = 5.09 km, *F* = 2.69 km; Fleming, 1997). We first subtracted open water and then calculated the proportional cover of oak and anthropogenic land within each individual bear use area per year (Baston, 2023). For individuals detected multiple years, we calculated the average cover across detected years. We compared general linear models and generalized additive models with a beta distribution and a logit link to determine how individual proportional diet was related to the proportion of anthropogenic landcover for mainland bears and to oak landcover for island bears (Wood, 2011, 2017). We determined the best model fit using Akaike information criterion (AIC; Burnham & Anderson, 2002).

### Vital rates

We tested demographic closure within six secondary sessions (in weeks) per primary period (in years) for mainland and island datasets (Otis et al., 1978). The islands were demographically closed, but the mainland violated closure (Appendix S3: Table S1). Therefore, we tested the mainland for closure during a truncated period—the first four secondary sessions within each primary period. This truncated period showed evidence of closure (Appendix S3: Table S1). To model apparent survival, recruitment, and population growth rate on the mainland and on the islands, we used a Huggins robust design reverse time Pradel model accounting for recapture probability and model selection bias (Nichols, 2016; Pradel, 1996). The robust design model harnesses a sampling structure which assumes the population is open to mortality and recruitment between primary periods, but geographically and demographically closed between secondary sessions. The Pradel model reverses capture histories so that per capita recruitment can be estimated. The parameters estimated reflect apparent recruitment and survival that can be influenced by migration. Due to the lack of a geographical barrier to prevent movement on and off our study area on the mainland, we also analyzed the mainland population using a Pradel model accounting for transient individuals (Telenský et al., 2024). This model includes a parameterization of residency probability, relative to the number of all individuals captured in each year (Appendix S3; Telenský et al., 2024).

We used individual identities to construct models that estimated 95% CI for abundance (*N̂*) and population growth rate (λ) at each primary sampling period; apparent survival (ϕ), recruitment (*f*) between primary periods; and conditional capture ($\hat{p}$) and recapture (*ĉ*) probabilities during secondary periods. We defined primary sampling periods as year (2020, 2021, 2022) and secondary sampling periods as six trapping sessions each summer. First, we ran a model set that included all bears (*n* = 133) grouped by location where they were detected (we censored three bears detected in both locations). We modeled capture and recapture probability, comparing models where detection varied by primary sampling period, secondary sampling period, by sex, location, or was constant. We compared models where capture probability and recapture probability had the same slopes and had different slopes. We used the top model (≤2 corrected AIC [AIC_c_]) for detection to determine covariate effects on survival and recruitment. We tested apparent survival for individual covariates including proportion of anthropogenic and oak landcover in bear use area (Wiscland2, Wisconsin Department of Natural Resources, 2016), proportion of human food and hard mast in diet, sex, time, and location (mainland or islands). To investigate population level covariate effects and avoid collinearity, we then modeled the mainland and islands separately. We used the same stepwise modeling framework, first determining nuisance parameter structure and then testing for covariate effects on survival and recruitment. For the mainland, we tested survival for individual covariates including anthropogenic landcover, proportion of human food and proportion of hard mast in diet, sex, and time. For island models, we included individual covariates for proportion of hard mast in diet, proportion oak landcover in bear use area (Vegetation Inventory and Map, Hop et al., 2010), sex, and time. We compared models where recruitment varied by time, sex, and was constant. Where we did not have a stable isotope sample for a bear (30% [*N* = 23] missing data for the mainland and 21% [*N* = 13] for the islands), we imputed missing values as zeros after centering covariates. Additionally, we ran island models excluding bears with missing stable isotope diet covariates (Appendix S3 for details). We derived bear abundance estimates for each island by adding location as a grouping variable to our top model. We calculated densities as abundance divided by island area (Basswood = 7.7 km^2^, Oak = 20.3 km^2^, Sand = 11.6 km^2^, Stockton = 40.0 km^2^) and mainland study area (62.2 km^2^). We used program *RMark* v3.0.0 (Laake, 2013) for all demographic modeling. We ranked models using AIC_c_ (Burnham & Anderson, 2002). We considered models within 2 AIC_c_ of the top model competitive and considered covariates as informative if 95% CI did not overlap zero (Arnold, 2010). Chat GPT 5.1 was used to assist with editing code and refining models. All code and outputs were validated by the authors.

## RESULTS

### Stable isotopes

**FIGURE 2:**
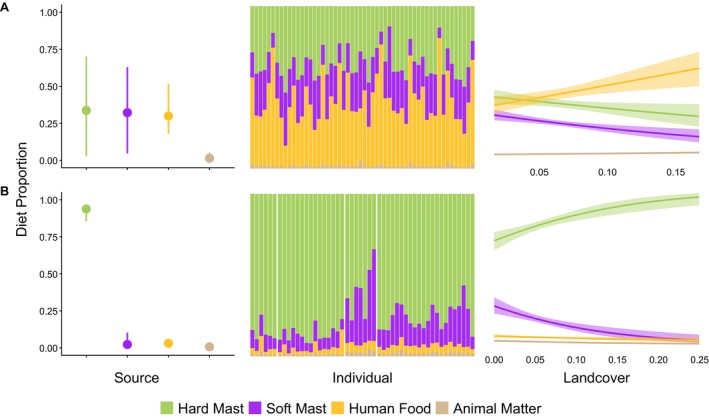
Diet estimates for black bears (*Ursus americanus*) including four source groups (soft mast [*Cornus* spp., *Ribes* spp., *Rubus* spp., *Vaccinium* spp., *Amelanchier arborea*, *Fragaria* spp., *Prunus* spp., *Aralia nudicaulis*, and *Sambucus canadensis*], hard mast [*Quercus* spp.], human food, and animal matter [ants and ant larvae; *Formicidae*, white tailed deer; *Odocoileus virginianus*, and beaver; *Castor canadensis*]) in (A) mainland Wisconsin (*n* = 56) and (B) the adjacent Apostle Islands National Lakeshore, Wisconsin, USA (*n* = 49). From left to right, population median and 95% credible intervals for proportional diet, mean individual proportional diet with gaps between bars separating bears grouped by islands (Basswood, Oak, Sand, and Stockton left to right), and generalized linear model showing the individual mean proportional diet as a function of percent landcover types (Mainland—anthropogenic landcover and islands—oak landcover) in bear use areas.

Bears on mainland Wisconsin, at the population level, consumed large amounts of human food (30% [18%, 51.5%]; Figure 2A), nearly equal parts hard mast (33.7%, 95% CI [2.8%, 70%]) and soft mast (32.2% [4.7%, 63%]), but minimal animal matter (1.4% [0.1%, 5.4%]). The individual bear with the highest reliance on human food consumed an average of 78.6% human food (13.2% hard mast, 6.4% soft mast, and 1.8% animal matter), while the mainland bear with the lowest reliance on human food consumed 13.6% human food (53.3% hard mast, 32.6% soft mast, and 0.6% animal matter).

The bear population on the Apostle Islands consumed almost entirely natural foods (Figure 2B). Notably, island bears consumed more hard mast (93.8% [85.5%, 96.3%]) than soft mast (2.3% [0.1%, 10.3%]) and minimal animal matter (0.7% [0%, 3.2%]) or human foods (3.1% [0.9%, 4.2%]).

### Landcover

The consumption of human food subsidies increased with the proportion of anthropogenic landcover within a bear use area (*R* ^2^_adj_ = 0.13, *F*_1,54_ = 9.4, *p* = 0.003). The proportion of hard mast consumed by island bears increased with the amount of oak landcover within a bear use area (*R* ^2^_adj_ = 0.29, *F*_1,48_ = 6.74, *p* < 0.001).

### Vital rates

Bear populations on the Apostle Islands and the mainland of Wisconsin exhibited notable differences in vital rates. The top model included the effect of location on survival (model weight = 0.53), recruitment differing by location, and detection probability differing by location and year (Appendix S2: Tables S1–S4). A second top model included the effect of hard mast on bear survival (model weight = 0.45; β = 5.15; 95% CI [3.04, 7.26]).

**FIGURE 3:**
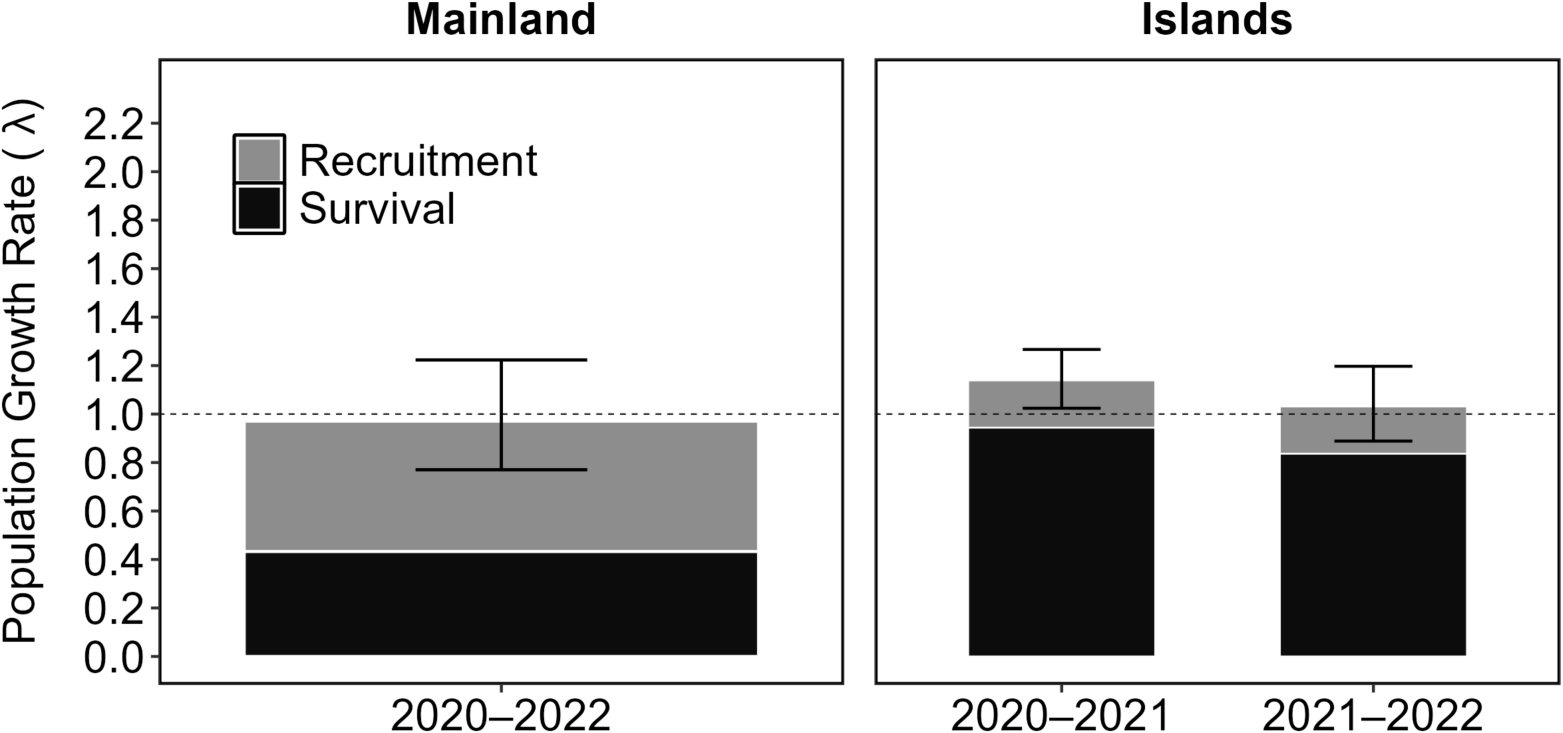
The population growth rate (λ) comprised of apparent recruitment and apparent survival of black bears (*Ursus americanus*) in the Apostle Islands and mainland Wisconsin, USA. Mainland Pradel model with transience included constant survival, constant recruitment, and probability of capture equal to probability of recapture and varying by week. Island Pradel models included survival varying by hard mast and year, constant recruitment, and probability of capture equal to probability of recapture and varying by week.

In mainland only models, hard mast was not correlated with survival. The top model for mainland bears included the effect of sex on survival (model weight = 0.48; Table 1), constant recruitment, and detection probability varying by year (Appendix S2: Tables S5 and S6). Apparent survival was higher for females (Φ = 0.62, 95% CI [0.41, 0.79]) than for males (Φ = 0.33, 95% CI [0.21, 0.49]). Overall, survival was 0.44 (95% CI [0.32, 0.56]) and recruitment was 0.54 (95% CI [0.33, 0.73]), resulting in λ = 0.98 (95% CI [0.77, 1.23]; Figure 3). Detection probability increased each year ($\hat{p}$_2020_ = 0.18, 95% CI [0.13, 0.25]; $\hat{p}$_2021_ = 0.36, 95% CI [0.29, 0.43]; $\hat{p}$_2022_ = 0.37, 95% CI [0.40, 0.44]). Bear abundance in the study area was 45.4 (95% CI [37.6, 64.8]) in 2020, 36.5 (95% CI [34.7, 43.4]) in 2021, and 40.6 (95% CI [38.7, 47.5]) in 2022. Density was between 0.56 and 0.73 bears/km^2^ (Appendix S2: Table S7). The resultant vital rate estimates of models containing 4 versus 6 secondary sessions on the mainland were nearly identical, and similar when accounting for transient bears (Appendix S3: Table S3).

**TABLE 1:** Akaike information criterion (AIC) model comparison for mainland Robust Design Pradel model.

Model	*K*	AIC_c_	ΔAIC_c_	Weight
Φ(~sex)	6	1042.22	0.00	0.48
Φ(~sex + year)	7	1043.73	1.52	0.23
Φ(~1)	5	1045.26	3.04	0.11
Φ(~HF)	6	1046.73	4.51	0.05
Φ(~anthro)	6	1046.88	4.66	0.05
Φ(~HM)	6	1047.30	5.08	0.04
Φ(~HF + year)	7	1048.69	6.47	0.02
Φ(~anthro + year)	7	1048.95	6.73	0.02
Φ(~HM + year)	7	1049.33	7.12	0.01

*Note*: Number of parameters (*K*), corrected AIC for small sample size (AIC_c_), difference in AIC_c_ (ΔAIC_c_), and model weight for models to determine covariate effects on black bear survival (*n* = 77, *Ursus americanus*) in mainland, Wisconsin, USA. Models ≤2 ΔAIC_c_ of top model were considered competitive models. Constant recruitment (*f* ~ 1) and capture probability constant and equal to recapture probability for all models (*p* = *c* ~ year).

Abbreviations: Φ, apparent survival; anthro, proportion of anthropogenic land in bear use area; HF, human food diet proportion; HM, hard mast diet proportion.

**FIGURE 4:**
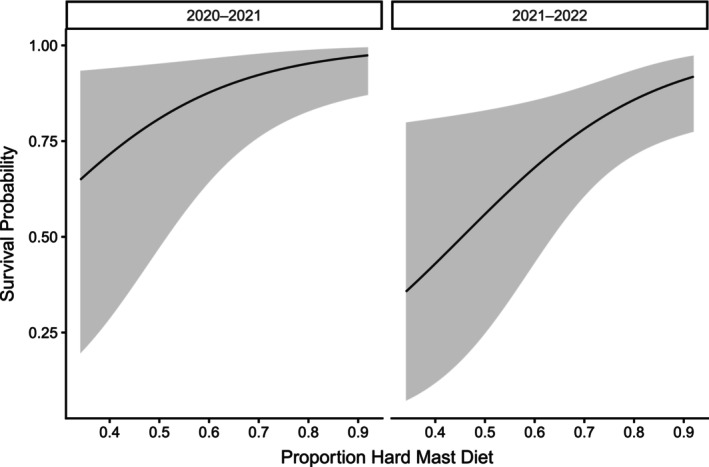
The proportion of hard mast within individual diet had a positive effect on survival of black bears (*Ursus americanus*) in the Apostle Islands Wisconsin, USA. Shaded area is 95% CI.

Bear survival in the Apostle Islands increased with the proportion of hard mast in diet (Figure 4; β = 5.35; 95% CI [0.63, 10.08]). The top model included the proportion of hard mast in diet and the effect of year on survival (model weight = 0.28; Table 2), constant recruitment, and detection probability varying by week (Appendix S2: Tables S8 and S9). Unlike the mainland, bear survival on the islands differed by year. Between 2020 and 2021, survival was 0.94 (95% CI [0.81, 0.99]), and recruitment was 0.19 (95% CI [0.11, 0.32]), resulting in λ = 1.14 (95% CI [1.03, 1.27]). Between 2021 and 2022 survival was 0.83 (95% CI [0.69, 0.92]) and recruitment was the same as in 2020–2021 resulting in λ = 1.03 (95% CI [0.89, 1.20]; Figure 3). Detection probability was highest the second secondary session ($\hat{p}$_1_ = 0.47, 95% CI [0.39, 0.55]; $\hat{p}$_2_ = 0.51, 95% CI [0.43, 0.59]; $\hat{p}$_3_ = 0.46, 95% CI [0.38, 0.54]; $\hat{p}$_4_ = 0.38, 95% CI [0.31, 0.47]; $\hat{p}$_5_ = 0.31, 95% CI [0.24, 0.39]; $\hat{p}$_6_ = 0.40, 95% CI [0.33, 0.49]). We identified two other competitive models: The second ranking model contained only the proportion of hard mast in diet and no difference in annual survival. In the third model (difference in AIC_c_ [ΔAIC_c_] = 1.48), apparent survival varied by the proportion of oak cover in a bear use area and year (model weight = 0.12). In this model, bear survival probability increased with the proportion of oak cover in bear use area, but the CI overlapped zero. On the Apostle Islands, bear densities ranged from 0.41 to 0.80 bears/km^2^ with average density across the islands being 0.61 bears/km^2^ in 2020, 0.59 bears/km^2^ in 2021, and 0.68 bears/km^2^ in 2022 (Appendix S2: Table S7).

**TABLE 2:** Akaike information criterion (AIC) model comparison for island Robust Design Pradel model.

Model	*K*	AIC_c_	ΔAIC_c_	Weight
Φ(~HM + year)	10	1336.37	0.00	0.26
Φ(~HM)	9	1336.66	0.29	0.22
Φ(~oak + year)	10	1337.85	1.48	0.12
Φ(~oak)	9	1338.46	2.09	0.09
Φ(~sex + year)	10	1338.65	2.27	0.08
Φ(~sex)	9	1338.71	2.34	0.08
Φ(~1)	8	1338.93	2.56	0.07
Φ(~year)	9	1339.02	2.65	0.07

*Note*: Number of parameters (*K*), Corrected AIC for small sample size (AIC_c_), difference in AIC_c_ (ΔAIC_c_), and model weight for models to determine covariate effects on black bear (*n* = 62, *Ursus americanus*) in the Apostle Islands, Wisconsin, USA. Models ≤2 ΔAIC_c_ of top model were considered competitive models. Constant recruitment (*f* ~ 1) and capture probability constant and equal to recapture probability for all models (*p* = *c* ~ 1).

Abbreviations: Φ, apparent survival; HM, hard mast diet proportion; oak, proportion of oak (*Quercus* spp.) landcover in a bear use area.

## DISCUSSION

Two populations of the same species in close proximity (<2 km apart) exhibited different vital rates. Recruitment was higher than survival on the mainland whereas recruitment was lower than survival on the islands. Consistent with our predictions, and despite these different vital rates, both populations exhibited population stability (i.e., λ ≈ 1). Changes in vital rates appeared to ultimately be related to the different landscapes they inhabited, which featured different food resource availability driving what bears in these populations consumed. As predicted, bears inhabiting the mainland consumed more human food than island populations. Mainland bears consumed 30% human foods at the population level, with some individual bears exhibiting a diet that was nearly 80% human foods. This contrasted with bears inhabiting the nearby protected islands, which consumed 3% human foods with some individuals consuming up to 10% human foods. Our population diet estimates of human food consumption for mainland Wisconsin bears were similar to other statewide estimates with bears consuming 43%–47% human foods (Kirby et al., 2017; Smith et al., 2025). This is higher than the food conditioned bear population in Yosemite National Park (35%) prior to human food abatement programs (Hopkins et al., 2014). The amount of human food consumed by bears increased with the human footprint, which has also been reported elsewhere in the United States (Kirby et al., 2019), as well as within Wisconsin (Smith et al., 2025). For example, bears with access to corn fields can have a diet consisting of 95% corn (Ditmer et al., 2016), whereas bears with little access to corn fields consume natural foods (Ditmer et al., 2016). These findings are similar to our observations where bears on protected islands consumed almost exclusively natural food (96.9%) compared to the bears with access to human food on the mainland. The flexible nature of bear foraging enables them to exploit a diversity of resources, including human foods like agricultural resources, human refuse, and hunter bait piles (Morin et al., 2024). Overall, when human foods enter the ecosystem, bears can switch their diet to incorporate human foods. Human foods can have profound effects on individuals, populations, species interactions, and community composition (Brunk et al., 2021; Newsome, Dellinger, et al., 2015). In contrast, the Apostle islands appeared to maintain a historical trophic pathway composed primarily of natural food items.

The population growth rates of bears in a protected island ecosystem, featuring little hunting or food subsidies, were primarily governed by survival. Notably, survival was a function of hard mast consumption. The importance of oak to bears has been well documented (Elowe & Dodge, 1989; Rogers, 1987), and population growth rates have previously been tied to oak mast (Obbard & Howe, 2008). Mast failure has been shown to increase cub mortality (Vaughan, 2002) and even lead to reproductive failure (Eiler et al., 1989; Rogers, 1976). Although bears will readily adjust their home ranges or change their diet (Bogdziewicz et al., 2016) to incorporate human foods in response to masting failure (Noyce & Garshelis, 1997), bears on the Apostle Island are relatively isolated (Cooper et al., 2024). Therefore, most bears are geographically constrained by the food resources that occurred within the island they inhabit. A decline in survival on the islands (2021–2022) coincided with a record low year for oak, hazel, and dogwood in the region (Rettler et al., 2021). We suspect that this mast failure contributed to lower survival, although a longer monitoring set would better elucidate this pattern. While food shortages have been linked to increased intraspecific conflict in bears elsewhere (Lindzey et al., 1986), the Apostle Islands exhibit some of the highest incidences of cannibalism in this species (Allen et al., 2022; Trauba, 1996). Such intraspecific conflict is likely an important driver behind bear survival in this population. The survival rates in this study were the same as between 1984 and 2001 (0.80; 95% CI [0.75, 0.86]; Gesch, 2003), when these incidences of cannibalism were recorded. Consequently, bears on the Apostle islands with little human food availability and no hunting appeared to be governed by naturally fluctuating resources eliciting strong density dependent mechanisms of population regulation.

Survival of bears inhabiting the mainland of Wisconsin did not appear sensitive to changing food availability. We did not detect an effect of mast, human food consumption, or landcover type on bear survival. Furthermore, bear survival on the mainland was constant across years and lower on the mainland compared to the islands. We attribute these patterns to several mechanisms. First, mainland bear vital rates could be decoupled from hard mast due to the presence of human foods buffering resource limitation. For example, during the low mast in 2020–2021, mainland bears may have relied on alternative food sources like crops, human refuse, or hunter bait piles to offset low natural food abundance. It is also possible that bears could simply travel to other areas if there is low food availability within their home range. Our apparent survival and recruitment estimates cannot separate the processes of birth from immigration nor death from emigration. Therefore, it is possible that differing vital rates between mainland and islands is a difference in movement patterns. Another possibility is that the decoupling of survival and diet may have been diluted by the effects of hunting. Hunting can elicit density dependent changes in recruitment patterns and the age of primiparity in bears and other species (Engen et al., 2014; Obbard & Howe, 2008; Servanty et al., 2011). In 2020 and 2021 combined, just over 200 bears were harvested by hunters in and around our mainland study area, including several bears relocated for nuisance complaints (i.e., from the Red Cliff Reservation and Wisconsin DNR Game Management Unit 03; Appendix S1: Table S1). Finally, the effects of human food subsidies and hunting may interact to drive bear vital rates. For example, in years of low food abundance, female bears visit hunter bait more frequently (Noyce & Garshelis, 1997), where they are more likely to be harvested. Given that it is illegal to harvest cubs and females with cubs in Wisconsin (Wisconsin Department of Natural Resources, 2019), the supplemental food from baiting may then lead to relatively higher reproductive rates (Stringham, 1990) but reduce adult survival. Changing reproductive patterns for bears on the mainland are supported by the observations that the age of primiparity is younger on the mainland (3.9 years) with females producing cubs every 2 years compared to the islands where age at primiparity is later (4.8 years) and females often skip breeding cycles (Fleming, 1997). Hunting and human food subsidies have both been shown to reduce the age of primiparity in bears elsewhere (Obbard & Howe, 2008; Wightman et al., 2022) and hunting can lead to compensatory recruitment (Servanty et al., 2011). Overall, we suggest that hunting increases mortality, and the subsequent increase of per capita food availability combined with the presence of human food subsidy is a mechanism allowing bears to account for losses via compensatory recruitment.

While our vital estimates could be influenced by permanent immigration, we are confident that our findings reflect important differences in the ecology of these two different populations. Islands were demographically closed, but the mainland violated closure. Nevertheless, when we reanalyzed the mainland with a truncated sampling window to satisfy closure, and when accounting for transient dispersers, our findings were the same: that bears on the mainland exhibited higher recruitment and lower survival compared to bears inhabiting the islands. Our results align with previous research that compared reproductive patterns on the islands compared to the mainland and found that bears on the mainland reproduced at an earlier age and with greater frequency (Fleming, 1997). Thus, our findings support the idea that these two neighboring bear populations are operating under different demographic processes.

Anthropization confers both risk and reward to animals, with some species winning and some losing based on life history strategies (Suraci et al., 2021). Studies on terrestrial mammals have shown that with anthropization, species with dietary diversity and faster reproductive strategies are favored (Santini et al., 2019; Suraci et al., 2021). Mammals that adapt to anthropogenic habitats tend to have larger litters, faster development times, and larger body sizes (Santini et al., 2019). Here, a trade‐off in vital rates may have been a response to anthropogenic landscapes, indicating a potential for adaptation to anthropogenic habitats to occur. While not all species will be able to exhibit such plasticity in vital rates leading to a trade‐off, this pattern may be applicable to species featuring fast life histories, precocial species that can have larger litters or reproductive effort earlier in life (Cayuela et al., 2022; Jones et al., 2008; Servanty et al., 2009), and species with dietary plasticity (Newsome & van Eeden, 2017). For example, wild boars (*Sus scrofa*) can accelerate generation times when facing high levels of human‐induced mortality and non‐limiting food resources (Servanty et al., 2011). Yet, most ungulates cannot increase reproductive effort to account for increases in adult mortality, so increases in predation or hunting can lead to population declines (Nilsen et al., 2009; Wittmer et al., 2005). For black bears, which can respond to human landscapes via altered vital rates, we suspect that there will be a point at which increased human subsidies will not be able to offset mortality rates or situations where human‐induced mortality is so high that increased recruitment will be unable to maintain viable populations (Lewis et al., 2014). We demonstrate altered vital rates in a large carnivore and suggest that human food subsidies and hunting are the mechanism. Therefore, it appears that foraging plasticity—and especially the ability to exploit human foods—can be a mechanism by which some animals can persist in anthropogenic landscapes via increased recruitment.

Protected areas and islands can function as refugia from human disturbance in a rapidly changing world (Bellemain & Ricklefs, 2008; Hoff et al., 2025; Morelli et al., 2020). The Apostle Islands have been identified as a refugia for native carnivores (Cooper et al., 2024; Druin et al., 2025; Smith et al., 2021). The islands, being relatively protected from anthropogenic disturbances including land use change and hunting, can support large populations but also appear to act as an important source of individuals for mainland populations (Cooper et al., 2024; Smith & Pauli, 2024). Our work suggests that these islands may not only act as a refugia for species protection but also for important ecological interactions. Since islands were protected from human subsidies, they retained historical trophic pathways. Specifically, bears on the islands consumed virtually only natural foods, and their vital rates appeared dependent on the consumption of native masting plants. By maintaining trophic pathways, bears on the islands retained their historical demographic strategies. Refugia, then, have the potential to maintain trophic interactions and natural ecosystem processes.

## AUTHOR CONTRIBUTIONS

Monica R. Cooper and Jonathan N. Pauli conceived the ideas and designed the methodology. Monica R. Cooper, Andrew Edwards, Kassandra Arts, and Ronald Nordin Jr collected the data. Monica R. Cooper and Jonathan N. Pauli analyzed the data. Monica R. Cooper and Jonathan N. Pauli led the writing of the manuscript. All authors contributed critically to the manuscript and gave final approval for publication.

## CONFLICT OF INTEREST STATEMENT

The authors declare no conflicts of interest.

## Supporting information


Appendix S1.

Appendix S2.

Appendix S3.

## Data Availability

Data and code (Cooper et al., 2026) are available on Figshare at https://doi.org/10.6084/m9.figshare.28914422.v2.
