# Supplementary material for: A trade‐off in vital rates for a large carnivore inhabiting an anthropogenic landscape and a protected island ecosystem
Source: Ecol Appl. 2026 Aug 2;36(5):e70289. doi: 10.1002/eap.70289 (PMC13430100; doi:10.1002/eap.70289)
Supplement: Supplementary file 1 — Appendix S1. [file EAP-36-e70289-s002.pdf]

Appendix S1

A trade-off in vital rates for a large carnivore inhabiting an anthropogenic landscape and a protected island ecosystem

Monica R. Cooper, Andrew Edwards, Kassandra Arts, Ronald Nordin Jr., Jonathan N. Pauli

Ecological Applications

Stable Isotopes

Results

Black bear diets calculated using a single trophic discrimination factor (TDF) for all sources were similar to the values reported with source specific TDFs (Table S3 and Figure S2). The only notable difference was in the mainland model, where population level diet proportion was 60% soft mast and 1% hard mast whereas with source specific TDF's hard mast was 34% and soft mast was 32%.

Table S1. The number of black bears (*Ursus americanus*) harvested by hunters (Hunter Harvest; total [males, females]) and relocated due to conflict with humans (Nuisance Relocations) for the Red Cliff Reservation, the Wisconsin DNR Game Management Unit 03 (GMU 03; 1,492 km^2), and the Apostle Islands National Lakeshore in 2020 and 2021. GMU 03 nuisance relocation data were not available (NA) in 2020.

**Location**	**Year**	**Nuisance Relocations**	**Hunter Harvest**
Red Cliff	2020	2	4 (2, 2)
Red Cliff	2021	1	3 (1, 2)
GMU 03	2020	NA	161 (76, 84)
GMU 03	2021	1	31 (22, 9)
Apostle Islands	2020	0	0
Apostle Islands	2021	0	0

Table S2. Mean, digestible concentrations (Conc), trophic discrimination factor (TDF), and sample size (N) of d13C and d15N used in Bayesian mixing models. Standard deviations are in parenthesis. Citations indicate the origin of Mean and/or TDF values.

Source	Mean C	Mean N	Conc C	Conc N	TDF C	TDF N	N	Citation
**Hard Mast**	-28.17 (1.30)	-2.91 (1.51)	0.52	0.01	3.4 (0.2)	2.4 (0.2)	31	TDF- Kirby et al. 2017
**Soft Mast**	-30.06 (2.10)	-2.00 (2.26)	0.45	0.02	3.4 (0.2)	2.4 (0.2)	35	TDF- Kirby et al. 2017
**Animal Matter**	-26.23 (1.37)	1.98 (1.19)	0.52	0.12	2.1 (0.1)	3.9 (0.3)	36	TDF- Kirby et al. 2017
**Bait**	-25.43 (1.6)	3.94 (0.65)	0.51	0.01	4.1 (0.3)	2.8 (0.2)	27	Kirby et al. 2017
**Human Food**	-20.0 (0.9)	5.5 (0.6)	0.53	0.07	2.5 (0.5)	3.5 (0.5)	37	Newsome et al. 2015
**Corn**	-11.89 (0.08)	6.98 (0.98)	0.46	0.02	1.4 (0.3)	2.4 (0.2)	24	Ditmer et al. 2016

Table S3. Median population diet proportions and 95% credible intervals (CI) from the stable isotope mixing model run with a single TDF mixed diet omnivore for black bears on mainland Wisconsin (TDF_C = 2.0 (0.2); TDF_N = 3.3 (0.2)) and a C_3 diet omnivore for black bears on the Apostle Islands (TDF_C = 4.3 (0.4); TDF_N = 3.2 (0.3); Stephens et al. 2023).

**Source**	**Population**	**Median**	**95% CI**
Human Food	Islands	0.016	0.001-0.033
Hard Mast	Islands	0.84	0.638-0.96
Soft Mast	Islands	0.127	0.008-0.325
Animal Matter	Islands	0.016	0.001-0.04
Human Food	Mainland	0.284	0.239-0.365
Hard Mast	Mainland	0.091	0.003-0.333
Soft Mast	Mainland	0.601	0.404-0.705
Animal Matter	Mainland	0.008	0-0.039

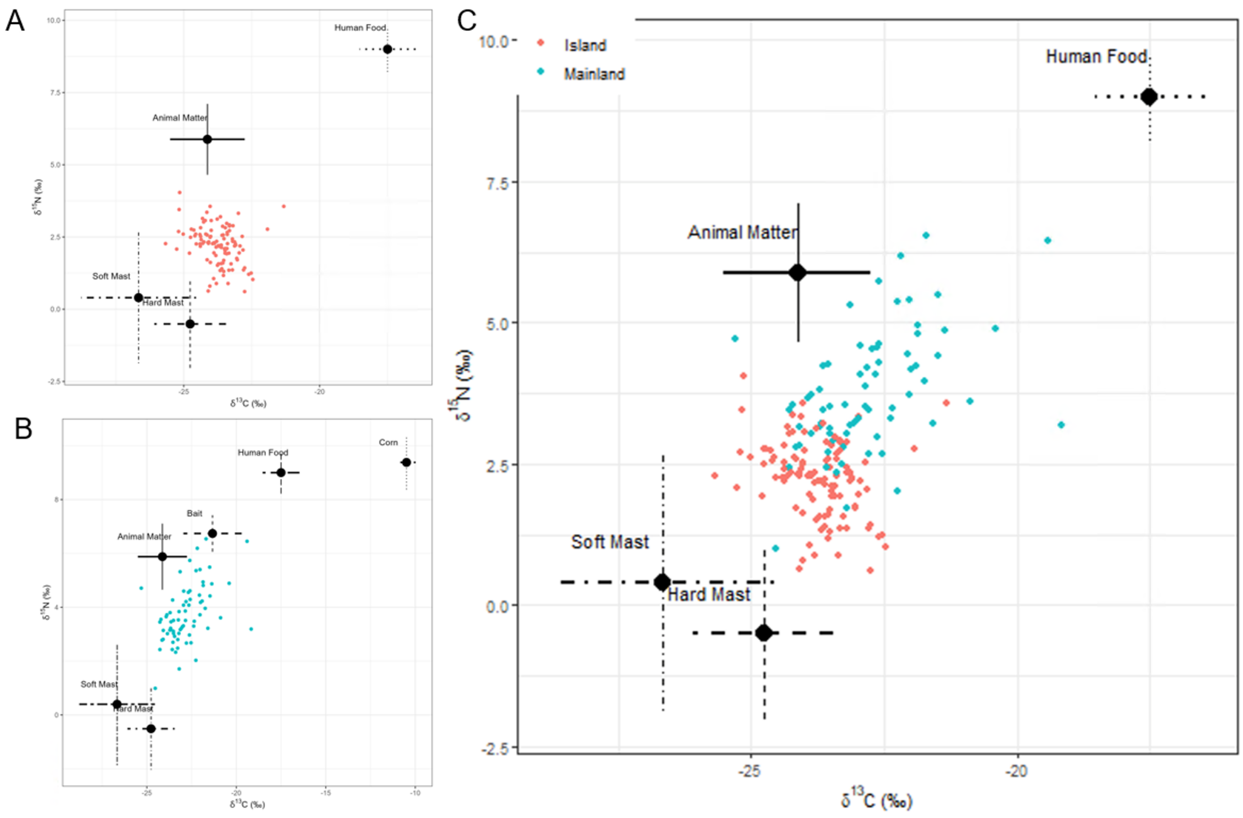

Figure S1. Stable isotope biplot displaying d13C and d15N (per mill) for black bears (*Ursus americanus*; n= 49) and sources including soft mast (berries, n=35, *Cornus* spp., *Ribes* spp., *Rubus* spp., *Vaccinium* spp., *Amelanchier arborea*, *Fragaria* spp., *Prunus* spp., *Aralia nudicaulis*, *Sambucus canadensis*), hard mast (acorns, n=33, *Quercus* spp.), animal matter (ants and ant larvae, n=13, *Formicidae*; white tailed deer, n=19, *Odocoileus virginianus*; and beaver, n=7, *Castor canadensis*), and human food (Newsome et al. 2015) in **A** Apostle Islands (n=49) and **B** mainland Wisconsin (n=56). Mainland Wisconsin mixing models included the sources listed above and, additionally, bait (Kirby et al. 2017) and corn (Ditmer et al. 2016). Human food, bait and corn were combined *a posteriori* in mainland model. **C** Both mainland and island bear biplot with sources from the island model. We did not run this model but display it here to show the samples in the same mixing space for direct comparison of the raw data.

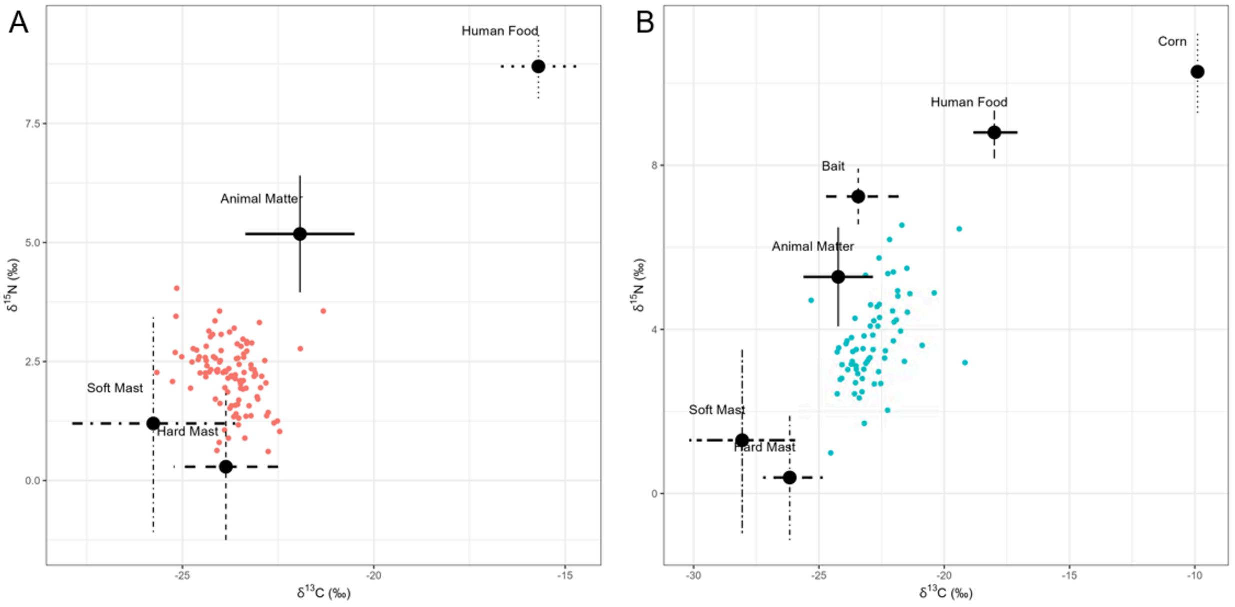

Figure S2. Biplots for stable isotope mixing models run with a single trophic discrimination factor (TDF) for all sources (Stephens et al. 2023). Stable isotope biplots displaying d13C and d15N (per mill) for black bears (*Ursus americanus*) and sources including soft mast (berries, n=35, *Cornus* spp., *Ribes* spp., *Rubus* spp., *Vaccinium* spp., *Amelanchier arborea*, *Fragaria* spp., *Prunus* spp., *Aralia nudicualis*, *Sambucus canadensis*), hard mast (acorns, n=33, *Quercus* spp.), animal matter (ants and ant larvae, n=13, *Formicidae*; white tailed deer, n=19, *Odocoileus virginianus*; and beaver, n=7, *Castor canadensis*), and human food (Newsome et al. 2015) in **A** Apostle Islands (n=49) included a TDF for C3 diet omnivores and **B** mainland Wisconsin (n=56) included a TDF for mixed diet omnivores. Mainland Wisconsin mixing models included the sources listed above and, additionally, bait (Kirby et al. 2017) and corn (Ditmer et al. 2016). Human food, bait and corn were combined *a posteriori* in mainland model.

References

Ditmer, M. A., Garshelis, D. L., Noyce, K. V., Haveles, A. W., & Fieberg, J. R. (2016). Are American black bears in an agricultural landscape being sustained by crops? *Journal of Mammalogy*, *97*(1), 54–67. https://doi.org/10.1093/jmammal/gyv153

Kirby, R., Macfarland, D. M., & Pauli, J. N. (2017). Consumption of intentional food subsidies by a hunted carnivore. *The Journal of Wildlife Management*, *81*(7), 1161–1169. https://doi.org/10.1002/jwmg.21304

Newsome, S. D., Garbe, H. M., Wilson, E. C., & Gehrt, S. D. (2015). Individual variation in anthropogenic resource use in an urban carnivore. *Oecologia*, *178*(1), 115–128. https://doi.org/10.1007/s00442-014-3205-2

Stephens, R. B., Shipley, O. N., & Moll, R. J. (2023). Meta-analysis and critical review of trophic discrimination factors ($\Delta^{13}C$ and $\Delta^{15}N$): Importance of tissue, trophic level and diet source. *Functional Ecology*, *37*(9), 2535–2548. https://doi.org/10.1111/1365-2435.14403
