# Supplementary material for: A trade‐off in vital rates for a large carnivore inhabiting an anthropogenic landscape and a protected island ecosystem
Source: Ecol Appl. 2026 Aug 2;36(5):e70289. doi: 10.1002/eap.70289 (PMC13430100; doi:10.1002/eap.70289)
Supplement: Supplementary file 2 — Appendix S2. [file EAP-36-e70289-s001.pdf]

Appendix S2

A trade-off in vital rates for a large carnivore inhabiting an anthropogenic landscape and a protected island ecosystem

Monica R. Cooper, Andrew Edwards, Kassandra Arts, Ronald Nordin Jr., Jonathan N. Pauli

Ecological Applications

Robust Design Pradel Models

Results

Table S1. Model name, number of parameters (K), Akaike's Information Criterion corrected for small sample size (AICc), ΔAICc, and model weight comparing Huggins Robust Design Pradel models to determine nuisance parameter structure for capture probability (p) and recapture probability (c) of black bears (n= 133, *Ursus americanus*) on the mainland and the Apostle Islands, Wisconsin, USA. All models held survival probability (Φ) and recruitment (f) constant.

**Model**	**K**	**AICc**	**ΔAICc**	**weight**
p(~location + year)c()	6	2312.68	0.00	0.88
p(~location + week)c()	9	2316.76	4.08	0.11
p(~location)c(~location)	6	2323.15	10.47	0.00
p(~location)c()	4	2326.80	14.12	0.00
p(~sex)c()	4	2332.24	19.57	0.00
p(~week)c(~week)	13	2332.95	20.27	0.00
p(year)c()	5	2334.58	21.91	0.00
p(~sex)c(~sex)	6	2336.10	23.42	0.00
p(year)c(year)	8	2337.58	24.91	0.00
p(~week)c()	8	2338.62	25.94	0.00
p(~1)c()	3	2348.74	36.06	0.00

Table S2. AIC model comparison for mainland Robust Design Pradel model. Number of parameters (K), Akaike's Information Criterion corrected for small sample size (AICc), ΔAICc, and model weight for models to determine covariate effects on black bear survival (n=133, *Ursus americanus*) on the mainland and the Apostle Islands, Wisconsin, USA. Φ= apparent survival, HM= hard mast diet proportion, HF= human food diet proportion, anthro= proportion of anthropogenic landcover in bear use area, sex = bear sex, and oak= proportion of oak landcover in bear use area. Models $\leq$2 ΔAICc of top model were considered competitive models. Recruitment constant (f ~1) and capture probability varying by location (mainland vs island) and year, and equal to recapture probability for all models (p=c ~ location + year).

**Model**	**K**	**AICc**	**ΔAICc**	**weight**
Φ(~location)	7	2289.30	0.00	0.53
Φ(~HM)	7	2289.64	0.34	0.45
Φ(~anthro)	7	2296.27	6.97	0.02
Φ(~HF)	7	2299.49	10.19	0.00
Φ(~sex)	7	2301.28	11.98	0.00
Φ(~oak)	7	2306.56	17.25	0.00
Φ(~1)	6	2312.68	23.37	0.00

Table S3. Model name, number of parameters (K), Akaike's Information Criterion corrected for small sample size (AICc), ΔAICc, and model weight comparing Huggins Robust Design Pradel models to determine covariate effects on recruitment for black bears (n=133, *Ursus americanus*) on the mainland and Apostle Islands, Wisconsin, USA. All models held survival probability (Φ) varying with location (mainland vs islands) and capture probability (p) equal to recapture probability (c) and varying with location and year.

**Model**	**K**	**AICc**	**ΔAICc**	**weight**
f ~ location	8	2275.83	0.00	1.00
f ~ sex	8	2289.19	13.36	0.00
f ~ 1	7	2289.30	13.47	0.00
f ~ year	8	2290.47	14.64	0.00

Table S4. Parameter estimates, lower and upper 95% confidence intervals (lcl, ucl) for survival (Φ), recruitment (f) population growth rate (λ) and detection probability (p) of black bears (n=133, *Ursus americanus*) detection on the Apostle Islands (IL) and mainland (ML) Wisconsin, USA. Estimates are from the top model including all bears, where survival varies by location, recruitment varies by location, and detection varies by location and year.

Parameter	estimate	lcl	ucl
Φ IL	0.89	0.80	0.95
Φ ML	0.45	0.33	0.58
f IL	0.16	0.08	0.28
f ML	0.61	0.37	0.80
λ IL	1.05	0.94	1.18
λ ML	1.06	0.85	1.32
p IL 2020	0.32	0.27	0.38
p IL 2021	0.47	0.42	0.52
p IL 2022	0.45	0.39	0.50
p ML 2020	0.23	0.18	0.28
p ML 2021	0.35	0.29	0.41
p ML 2022	0.33	0.28	0.38

Table S5. Model name, number of parameters (K), Akaike's Information Criterion corrected for small sample size (AICc), ΔAICc, and weight comparing Huggins Robust Design Pradel models to determine nuisance parameter structure for capture probability (p) and recapture probability (c) of black bears (n= 77, *Ursus americanus*) in mainland Wisconsin, USA. All models held survival probability (Φ) and recruitment (f) constant.

**Model**	**K**	**AICc**	**ΔAICc**	**weight**
p(~year)c()	5	1045.26	0.00	0.64
p(~year)c(~year)	8	1048.26	3.00	0.14
p(~sex)c()	4	1048.51	3.25	0.13
p(~sex)c(~sex)	6	1050.34	5.08	0.05
p(~week)c()	8	1051.58	6.32	0.03
p(~week)c(~week)	13	1055.48	10.22	0.00
p(~1)c()	3	1057.64	12.37	0.00
p(~1)c(~1)	4	1059.28	14.02	0.00

Table S6. Model name, number of parameters (K), Akaike's Information Criterion corrected for small sample size (AICc), ΔAICc, and weight comparing Huggins Robust Design Pradel models to determine covariate effects on recruitment for black bears (n=77, *Ursus americanus*) in mainland, Wisconsin, USA. All models held survival probability (Φ) varying with sex and capture probability (p) equal to recapture probability (c) and varying with year.

**Model**	**K**	**AICc**	**ΔAICc**	**weight**
f(~1)	6	1042.22	0.00	0.43
f(~year)	7	1042.28	0.06	0.42
f(~sex)	7	1044.31	2.09	0.15

Table S7. Abundance derived from the top models for survival of black bears (*Ursus americanus*) on four islands in the Apostle Islands National Lakeshore and mainland Wisconsin, USA in 2020-2022. Density (bears/km^2; Basswood = 7.7 km^2, Oak = 20.3 km^2, Sand = 11.6 km^2, Stockton = 40.0 km^2, mainland 62.2 km^2) with lower and upper confidence intervals in parentheses.

Island	Year	Abundance	Density
Basswood	2020	5.2 (5.0- 7.7)	0.65 (0.63-0.96)
Basswood	2021	5.2 (5.0- 7.7)	0.65 (0.63- 0.96)
Basswood	2022	6.2 (6.0- 8.9)	0.78 (0.75- 1.10)
Oak	2020	13.5 13.1- 17.0)	0.66 (0.64- 0.83)
Oak	2021	16.6 (16.1- 20.3)	0.80 (0.78- 0.99)
Oak	2022	15.6 (15.1-19.2)	0.76 (0.73- 0.93)
Sand	2020	8.3 (8.0-11.3)	0.70 (0.67- 0.95)
Sand	2021	6.2 (6.0- 8.9)	0.52 (0.50- 0.74)
Sand	2022	8.3 (8.0- 11.3)	0.70 (0.67- 0.95)
Stockton	2020	16.6 (16.1- 20.3)	0.41 (0.40- 0.50)
Stockton	2021	22.8 (22.1- 26.9)	0.56 (0.54- 0.66)
Stockton	2022	20.7 (20.1- 24.7)	0.51 (0.49- 0.61)
Mainland	2020	45.4 (37.6- 64.8)	0.73 (0.60- 1.00)
Mainland	2021	36.5 (34.7- 43.4)	0.56 (0.53- 0.66)
Mainland	2022	40.6 (38.7- 47.5)	0.65 (0.62- 0.76)

Table S8. Model name, number of parameters (K), Akaike's Information Criterion corrected for small sample size (AICc), ΔAICc, and model weight comparing Pradel models to determine nuisance parameter structure for capture probability (p) and recapture probability (c) of black bears (n= 62, *Ursus americanus*) in the Apostle Islands, Wisconsin, USA. Survival (Φ) and recruitment (f) held constant (~1) for all models. We excluded models that did not converge in downstream modeling (p~week, c~week).

Model	K	AICc	ΔAICc	weight
p(~week)c()	8	1338.93	0	0.76
p(~year)c()	5	1342.54	3.61	0.1
p(~sex)c()	4	1344.53	5.60	0.05
p(~1)c()	3	1344.92	5.99	0.04
p(~1)c(~1)	4	1346.96	8.03	0.01
p(~year)c(~year)	8	1347.41	8.48	0.01
p(~sex)c(~sex)	6	1348.64	9.71	0.00

Table S9. Model name, number of parameters (K), Akaike's Information Criterion corrected for small sample size (AICc), ΔAICc, and weight comparing Pradel models to determine whether recruitment (f) of black bears (n= 62, *Ursus americanus*) in the Apostle Islands, Wisconsin, USA is constant or varies by sex or year. Probability of capture (p) equal to recapture (c) and varying by week, and survival (Φ) varying by hard mast diet proportion (HM) and year for all models.

**Model**	**K**	**AICc**	**ΔAICc**	**weight**
f(~1)	10	1336.22	0.00	0.46
f(~sex)	11	1337.06	0.84	0.30
f(~ year)	11	1337.53	1.31	0.24
