# Supplementary material for: A trade‐off in vital rates for a large carnivore inhabiting an anthropogenic landscape and a protected island ecosystem
Source: Ecol Appl. 2026 Aug 2;36(5):e70289. doi: 10.1002/eap.70289 (PMC13430100; doi:10.1002/eap.70289)
Supplement: Supplementary file 3 — Appendix S3. [file EAP-36-e70289-s003.pdf]

Appendix S3

A trade-off in vital rates for a large carnivore inhabiting an anthropogenic landscape and a protected island ecosystem

Monica R. Cooper, Andrew Edwards, Kassandra Arts, Ronald Nordin Jr., Jonathan N. Pauli

Ecological Applications

Missing covariates, closure, and Pradel robust design with transience

Methods

Missing diet covariates

Where we did not have a stable isotope sample for a detected bear (21% [N = 13] for the islands), we excluded individuals from analysis. We first determined capture and recapture probability structure and then compared models for survival and recruitment, comparing the same model sets as the main analysis.

Closure

Because the closure test is sensitive to individual heterogeneity eliciting type I errors (Chao and Huggins 2005) we used $\alpha = 0.01$.

Pradel with transience

We extended the Pradel with transience model to include the robust design for detection and re-parameterized to directly estimate recruitment (Telenský et al. 2024). We fit the same model as the top model from our main analysis, which included survival varying by sex, constant recruitment, and capture probability equal to recapture probability and varying by year to estimate parameters and 95% credible intervals (CI's). We ran the model in BUGS using Nimble (de Valpine et al. 2017) with three parallel chains of 200,000 iterations, thinning to every 40th iteration and discarding the first 80,000 as burn-in.

Results

Missing diet covariates

Despite removing individuals with missing diet covariates, the top model for island bears included the effect of hard mast on survival. A second competitive model included the effect of hard mast and time (Appendix S2: Table S2). Bear survival increased with the proportion of hard mast in bear diet (6.36 95% CI [1.13, 11.60]).

Table S1. Otis test for demographic closure for black bears in mainland, Wisconsin (n=77, *Ursus americanus*) and in the Apostle Islands (n= 62), Wisconsin, USA. Tests compare the number of secondary sessions included (j; out of six) and displayed are p values (α = 0.01) for the test conducted within each year (2020-2022).

**Location**	**j**	**2020**	**2021**	**2022**
Mainland	6	0.70	0.01	0.002
Mainland	4	0.93	0.05	0.39
Islands	6	0.48	0.84	0.05

Table S2. Model comparison where bears with missing diet covariate data (n= 13) were excluded from analysis. Model name, number of parameters (K), Akaike's Information Criterion corrected for small sample size (AICc), Delta AICc (ΔAICc), and model weight comparing Pradel models to determine covariate effects on survival of black bears (n= 49, *Ursus americanus*) in the Apostle Islands, Wisconsin, USA. Covariates included the proportion of hard mast in bear diet (HM), the proportion of oak landcover in bear use area (oak), sex, and year. Probability of capture equal to recapture and varying by week (p=c ~ week), and constant recruitment (f ~1) for all models.

**Model**	**K**	**AICc**	**ΔAICc**	**weight**
Φ(~HM)	9	1110.73	0.00	0.37
Φ(~HM + year)	10	1111.38	0.65	0.27
Φ(~oak)	9	1113.17	2.45	0.11
Φ(~oak + year)	10	1113.79	3.06	0.08
Φ(~1)	8	1113.87	3.14	0.08
Φ(~year)	9	1114.89	4.16	0.05
Φ(~sex)	9	1115.64	4.92	0.03
Φ(~sex + year)	10	1116.64	5.92	0.02

Table S3. Models comparing three different modeling frameworks for black bears (n=77, *Ursus americanus*) on mainland, Wisconsin, USA. Parameters include survival (Φ), recruitment (f) population growth rate (λ), detection probability (p), and seniority. Models (Φ ~ sex, f~1, p=c ~year**)** compared are Huggins robust design Pradel models with four (RDP 4; parameter estimate (95% confidence interval)) and six (RDP 6; parameter estimate (95% confidence interval)) secondary sessions per summer and a robust design Pradel model accounting for transience including six secondary sessions per summer (RDPT 6; parameter estimate (95% credible interval)).

**Parameter**	**RDP 4**	**RDP 6**	**RDPT 6**
Φ	0.47 (0.34, 0.61)	0.44 (0.32, 0.56)	0.56 (0.42, 0.70)
f	0.51 (0.29, 0.73)	0.54 (0.29, 0.73)	0.46 (0.23, 0.73)
λ	0.98 (0.76, 1.26)	0.98 (0.78, 1.23)	1.00 (0.78, 1.33)
p 2020	0.24 (0.16, 0.35)	0.18 (0.13, 0.25)	0.22 (0.15, 0.32)
p 2021	0.46 (0.36, 0.56)	0.36 (0.29, 0.43)	0.36 (0.22, 0.58)
p 2022	0.43 (0.34, 0.53)	0.37 (0.30, 0.44)	0.37 (0.18, 0.68)
seniority	NA	NA	0.55 (0.41, 0.69)

References

Chao, A., & Huggins, R. M. (2005). Modern Closed-population Capture–Recapture Models. In S. C. Amstrup, T. L. McDonald, & B. F. J. Manly (Eds.), *Handbook of Capture-Recapture Analysis* (pp. 58–87). Princeton University Press. https://www.jstor.org/stable/j.ctt7sdj6.9

de Valpine, P., D. Turek, C.J. Paciorek, C. Anderson-Bergman, D. Temple Lang, and R. Bodik. 2017. Programming with models: writing statistical algorithms for general model structures with NIMBLE. *Journal of Computational and Graphical Statistics* 26:403-413. https://doi.org/10.1080/10618600.2016.1172487.

Telenský, T., Storch, D., Klvaňa, P., & Reif, J. (2024). Extension of Pradel capture–recapture survival-recruitment model accounting for transients. Methods in Ecology and Evolution, 15(2), 388–400. https://doi.org/10.1111/2041-210X.14262
